# Factors underlying bird community assembly in anthropogenic habitats depend on the biome

**DOI:** 10.1038/s41598-022-24238-x

**Published:** 2022-11-17

**Authors:** Anahí S. Vaccaro, Julieta Filloy

**Affiliations:** Departamento de Ecología, Genética y Evolución, IEGEBA (CONICET-UBA), Facultad de Ciencias Exactas y Naturales, Universidad de Buenos Aires, Ciudad Universitaria, Pabellón 2, Piso 4, C1428EGA CA Buenos Aires, Argentina

**Keywords:** Ecology, Environmental sciences

## Abstract

Production activities drive the replacement of original habitats with artificial ones, leading to new bird assemblages. In this study, we assessed if anthropogenic habitats acted as environmental filters causing functional redundancy or as promoters of functional divergence, depending on the biome. We also investigated if functional patterns derived from phylogenetic convergence or clustering. For this purpose, we computed the standardized effect sizes (SES) for avian functional and phylogenetic diversity using null models and compared the SES values among tree plantations, urban settlements (US), cattle pastures (CP), crop fields (CF) and natural habitats from two biomes: grassland and forest. We used generalized least squares models to test if functional and phylogenetic SES indicated functional redundancy or divergence, and phylogenetic convergence or clustering. We found functional redundancy in grassland and functional divergence in forest associated with environmental filtering and competitive exclusion, respectively. In grassland, functional structure was not associated with a clear phylogenetic pattern, while in forest functional divergence was caused by evolutionary convergence in CF and CP and conservation in US. The prevalences of functional redundancy and functional divergence patterns and their associated predominant mechanism of community assembly were found to depend on the biome and the regional species pool.

## Introduction

The intensification of production activities induces the replacement of original habitats by anthropogenic ones worldwide, leading to the formation of new community assemblages with varying relative abundances in different biomes. In highly modified environments (i.e., under high levels of environmental harshness) abiotic constraints are the main factor determining species establishment^1^. Abiotic conditions and resources may operate as a selective force against species unable to enter or persist in a community. Thus, environmental filtering is usually the predominant mechanism underlying assembly processes in anthropogenic habitats^2,3^. Species that persist in a given habitat exhibit tolerance to environmental conditions, which is determined by the functional trait composition of the community. As a result, co-existing species in the regional pool are functionally more similar than expected by chance (functional redundancy)^4^.

The resulting species and functional trait composition in communities from anthropogenic habitats may vary in response to land use across biomes, as it can alter the natural environmental filters imposed on the different species from the regional species pool^5,6^. For example, the loss of native taxonomic and functional diversity is greater for land uses involving drastic changes in vegetation structure than for those preserving it^3,7–9^. In this regard, anthropogenic habitats such as crop fields, tree plantations and cattle pastures may show different environmental filters according to the biome.

Studies conducted by our research group addressed the effect of different types of anthropogenic habitats located in contrasting biomes on bird species and functional trait assemblages. Results indicated a greater loss of native diversity in habitats that were environmentally different from their natural habitat. Thus, Vaccaro et al.^10^ reported that, in grassland, land conversion into tree plantations or urbanized areas led to higher loss of native bird species than conversion into cattle pastures and some types of agricultural lands. Likewise, Vaccaro and Bellocq^11^ found that open habitat types and tree plantations were the worst and best habitats for the conservation of bird species and functional trait assemblages of the native forest, respectively. In regard to biomes, environmental similarity between anthropogenic and native habitats has been emphasized as a key factor for preserving the taxonomic and functional diversity of bird assemblages in tropical forests^12,13^ and grasslands^10,14^.

In our previous studies mentioned above, some issues remain unexplored. Therefore, in this work we deepen the understanding of processes and factors underlying bird community assembly in distinct anthropogenic habitat types located in contrasting biomes. To this aim, we addressed the question of whether anthropogenic habitats act as environmental filters leading to functional redundancy, or as promoters of functional divergence, depending on the biome.

The inclusion of several attributes of biological communities in studies focused on the formation of assemblages in anthropogenic habitats provides meaningful information for more efficient conservation planning^15^. Here, we considered phylogenetic diversity, which is defined as the amount of evolutionary history represented in the species of a particular community^16^. It is used as a complement to taxonomic and functional diversities in community ecology studies on assembly processes^17,18^. Phylogenetically diverse communities are likely to be more resilient to environmental changes and to better preserve unique lineages^16,19^. As a result of environmental filtering, phylogenetic structure within communities may reflect either phylogenetic clustering or evenness depending on whether ecologically relevant traits are phylogenetically conserved or convergent^20^. Phylogenetic clustering occurs when species have a tendency to retain ancestral ecological characteristics allowing them to cope with environmental constraints ^20,21^. The phylogenetic structure is characterized by the co-occurrence of more closely related species than expected by chance. Moreover, environmental filtering can generate a uniform phylogenetic structure (phylogenetic evenness) if species share important traits to habitat specialization by evolutionary convergence (i.e., traits are labile and close relatives present traits suitable for different environments)^22–24^. The quantification of functional and phylogenetic structures of assemblages in anthropogenic habitats allows us to identify factors associated with environmental filtering (coexistence of ecologically similar taxa) or competitive exclusion^25^. This will depend on the biome where the land use type is located because species pools and the structure of assemblages in contrasting biomes such as forests and grasslands are expected to be shaped by different ecological, evolutionary and historical mechanisms^26^.

The purpose of this study was to test the existence of functional redundancy in different anthropogenic habitat types, which is the pattern predicted by the environmental filtering hypothesis when environmental filters are the main community assembly drivers. Taking into account that the transformation level of anthropogenic habitats depends on the biome, in this study we included four anthropogenic habitat types (tree plantation, urban settlement, cattle pasture and crop field) and natural habitats in two contrasting biomes: grassland and forest. Under the environmental filtering hypothesis, we expected a higher functional redundancy in habitat types showing a simpler vegetation structure than that found in the natural habitat (e.g. open habitat types in grassland and forest, tree plantations in the forest), due to the loss of specialist species. In line with this reasoning, we predict the occurrence of functional divergence in anthropogenic habitats with structural or environmental dimensions that were absent in the original environment (urban settlements in grassland and forest, tree plantations in grassland), due to the inclusion of species with new traits. Furthermore, we tested whether functional redundancy or divergence are due to phylogenetic evenness or phylogenetic clustering, since these imply different consequences for species assemblages in terms of traits favoring species persistence in anthropogenic habitats. For example, environmental filtering will have a more negative effect on phylogenetic clustering (i.e., loss of species from a unique linage) than on phylogenetic evenness (presence of species from different lineages).

## Methods

### Study design

**Figure 1:**
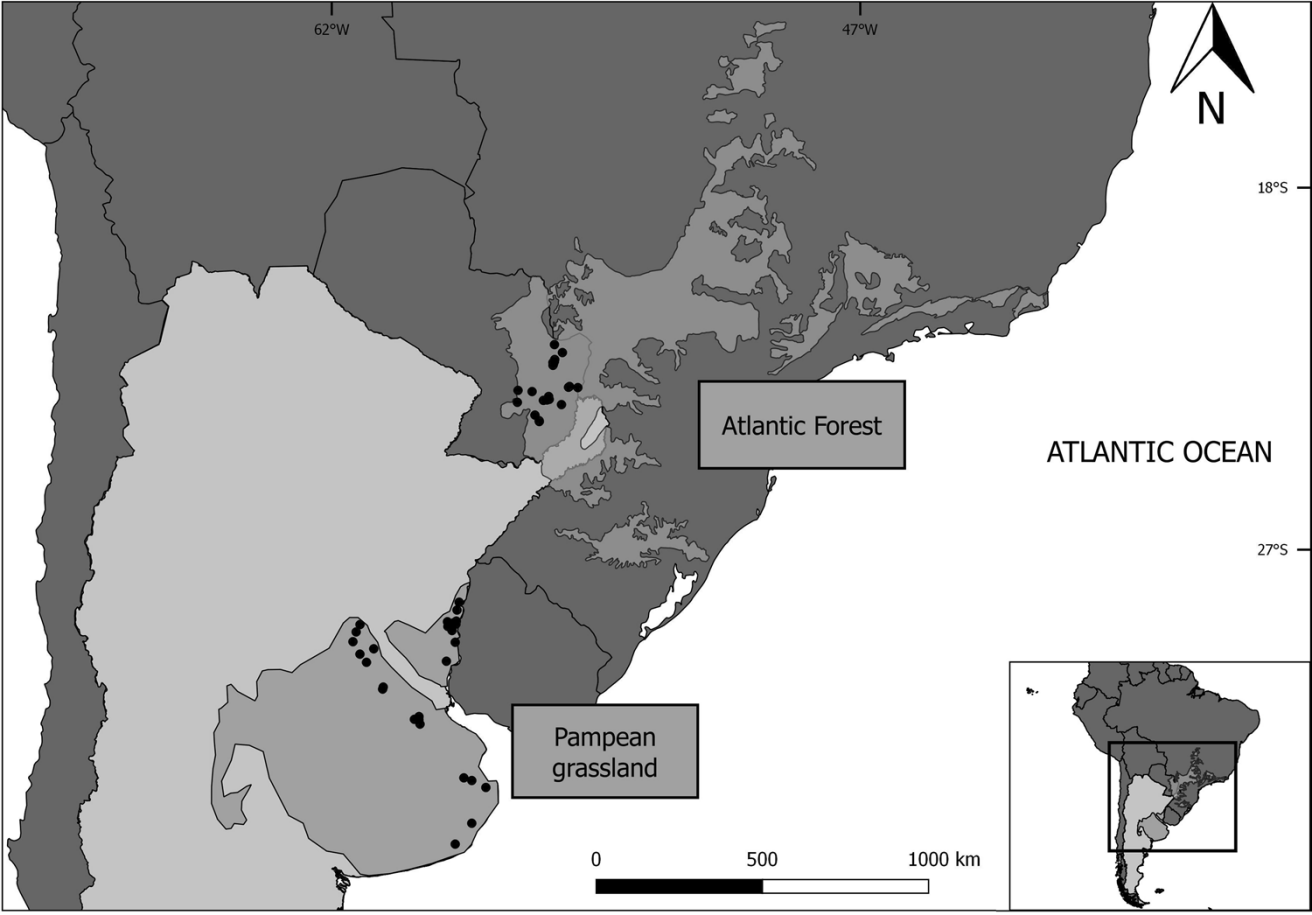
Location of the 27 sampling sites in the Pampean grassland (Grassland biome) and 22 sites in the Atlantic Forest (Forest biome). This map was generated with QGIS (3.28 Firenze version), a free and open-source Geographic Information System (https://www.qgis.org/).

We analyzed the occurrence of functional redundancy or divergence in bird assemblages from different anthropogenic habitat types located in two contrasting biomes to determine the potential mechanisms driving their formation. Moreover, we studied the phylogenetic structure of bird assemblages to identify phylogenetic clustering or evenness (overdispersion). For this purpose, we used null models to estimate the standardized effect sizes (SES) associated with functional and phylogenetic diversity of avian communities in anthropogenic habitat types and natural habitats. Data were obtained from birds sampled in natural habitats (NH) and four types of anthropogenic habitats (i.e., cattle pasture (CP), crop field (CF), tree plantation (TP) and urban settlement (US) located in the grassland and forest). We selected 27 study sites in the grassland comprising six sites per anthropogenic habitat type and three NHs, and 22 sites in the forest consisting of five CPs, five CFs, five TPs, four USs and three NHs (Fig. 1).

### Study area and selection of survey sites

We selected two contrasting biomes considered of priority importance for biodiversity conservation because they contained only small remnants of natural habitat due to extensive production activities^27,28^. These were the Pampean grassland and the subtropical Atlantic Forest (hereafter referred to as Grassland and Forest, respectively) in South America (Fig. 1). The Pampean grassland is located in central-eastern Argentina and covers approximately 444,990 km^2^. It has a temperate climate, with mean annual temperature ranging between 13 and 17 °C. This biome was originally dominated by grasses such as *Paspalum*, *Axonopus*, *Stipa*, *Bromus* and *Piptochaetium*^29^, and lacked trees. The Pampean region is at greatest risk due to agricultural expansion^30^. The Atlantic forest is located in southern Brazil, northeastern Argentina, and southern and eastern Paraguay^31^. The climate is subtropical and the original vegetation was a semi-deciduous forest^32^. Particularly in Paraguay, the Atlantic Forest has undergone large-scale deforestation for soybean production, livestock pastures and tree plantations. Indeed, only 17% of its original cover has been estimated to remain in isolated protected areas and private lands^31^.

### Bird surveys

Bird surveys were carried out in 2014 and 2015 during the breeding season (September to November), using the point-count technique with a fixed 50-m radius^33,34^. We established 10 observation points at each study site, which were located at least 150 m apart to minimize double-counting^35^. These were visited once on sunny days with calm winds. At each observation point, all birds seen or heard were identified simultaneously but independently by two trained observers during 5 min from sunrise to 4 h later; birds flying overhead were excluded. Due to the regional scale of the study we maximized the number of point counts instead of the time spent at each sampling point^36,37^. All bird songs were recorded using a digital recorder (Zoom H4next Handy Recorder) at all observation points during the 5-min period to ensure a reliable identification and to account for differences in visual detectability between habitat types^36^.

After the field work, birds' identification was accomplished by comparing our 490 bird songs recorded with published recordings^38^. We estimated species abundance at each study site by pooling the results obtained from the 10 observation points. Abundance data were used to build two site-by-species matrices for each biome: one including all species recorded and the other the regional species pool. The species in the regional pool were extracted from the regional species pool matrix (see below).

### Functional and phylogenetic diversity

To determine functional diversity, we first created a species-by-trait matrix separately for each biome. To construct this matrix we selected ecological traits (e.g., number of habitats used, sensitivity to human disturbance) and life-history traits (e.g., diet, clutch size and body mass), making a total of 11 traits (and their categories, see Supplementary Table S1). We followed the usual protocol for standardization of the trait matrix^39^ so that all traits were treated as categorical, and each category was binary. Each trait category was assigned 0 or 1 depending on whether it was present or not^10^. All trait categories were mutually exclusive (only one category of each trait was 1), except for diet, foraging substrate and nesting habitat. The resulting matrix for each biome was used to generate the regional species pool matrix. For the Grassland matrix, we selected species showing functional traits related to grassland or open habitats, such as foraging on the ground or nesting in grasses. For the Forest matrix, we considered species that use trees or shrubs for nesting, foraging or hiding.

To estimate phylogenetic diversity, we used a global phylogeny of birds (http://birdtree.org/)^40^ to create two phylogenetic trees for each biome: one for all species recorded and the other for the regional species pool. The consensus trees were obtained with the "consensus.edges" function in the R "phytools" package^41,42^.

### Data analysis

For the Grassland and the Forest we constructed a site-by-species matrix to estimate species abundance, a species-by-trait matrix to estimate functional diversity and a cophenetic distance matrix to estimate phylogenetic diversity. Each of them was performed with data from all the species recorded and the regional species pool, summing up a total of six matrices per biome.

To gain methodological consistency, we estimated functional and phylogenetic diversity with the mean pairwise distance (mpd) using the “mpd” function in the “picante” package of R^42,43^. Functional and phylogenetic mpd values were calculated for each site using sites-by-species, species-by-traits and cophenetic distance matrices built with the regional species pool.

To analyze functional and phylogenetic structures, we computed the standardized effect sizes (SES) for avian functional and phylogenetic diversity using null models and compared the SES values among habitat types for the two biomes. The SES values were estimated using the “ses.mpd” function in the “picante” package of R^42,44^. This function uses functional and phylogenetic mpd matrices as inputs to functional and phylogenetic SES, respectively. SES describes the difference between functional or phylogenetic distances in the community observed (*mpd.obs*) and the mean of null communities created with the randomization method (*mpd.rand.mean*), divided by the standard deviation of functional or phylogenetic distances in the null data (*mpd.rand.sd*). The randomization method used for null communities was ‘taxa.labels’, which shuffles distance matrix labels across all included in the distance matrix^42,43^. We used matrices of all the species recorded in each habitat type to estimate the functional and phylogenetic *mpd.obs*. Conversely, we used the regional species pool matrices to estimate the functional and phylogenetic mpd in null communities (*mpd.rand.mean* and *mpd.rand.sd*, respectively), because these species are expected to occur if the biome is not altered. We calculated a single SES value per site.

We fitted generalized least squares (gls) models with SESs values (response) and habitat type (factor), with both functional and phylogenetic values in each biome. Models were fitted using the “gls” function in the “nlme” R package^45^. In case of heteroscedasticity, we used the “varIdent” function with “nlme” package, which allows different variances per treatment. Then, with the “emmeans” function in the “emmeans” package^46^ we computed estimated marginal means for the specified factor (i.e., habitat type in this study) using the linear model as input (gls). The “emmeans” function allowed obtaining 95% confidence intervals (CI) for SES estimations of habitat types in each biome. Thus, we corroborated if zero was included in those CIs. Negative values of functional SES (i.e., both limits of CIs are negative) indicate that species are more similar in terms of functional traits than expected by chance (functional redundancy), suggesting that environmental filtering is the predominant assembly process. Conversely, positive values (i.e., both limits of CIs are positive) indicate that species are less similar than expected by chance (functional divergence), suggesting a possible competitive exclusion among assemblages^47^. With respect to phylogenetic structures, positive phylogenetic SES values indicate a greater phylogenetic distance between co-occurring species than expected by chance (phylogenetic evenness or overdispersion), while negative SES values may imply a smaller phylogenetic distance between species than expected (phylogenetic clustering). Additionally, with “emmeans” function we compared mean SES values between habitat types through Tukey multiple comparisons.

## Results

In the Grassland, we recorded a total of 2773 individuals belonging to 92 species: 333 individuals of 46 species in natural habitats, 787 individuals of 50 species in cattle pastures, 392 individuals of 33 species in crop fields, 360 individuals of 34 species in tree plantations, and 901 individuals of 24 species in urban settlements. Among the unclassified species of the regional pool, we found 3 in CP, 11 in TP, 3 in US and 2 in CF. The most abundant species in US were *Columba livia* (Rock pigeon) and *Passer domesticus* (House sparrow). In TP, there were species using trees or shrubs for foraging or nesting, such as *Leptotila verreauxi* (White-tipped dove), *Colaptes melanochloros* (Green-barred woodpecker) and *Lepidocolaptes angustirostris* (Narrow-billed woodcreeper) (see Supplementary Table S2).

In the Forest, we recorded a total of 2162 birds belonging to 150 species: 276 individuals of 64 species in natural habitats, 549 individuals of 69 species in cattle pastures, 246 individuals of 31 species in crop fields, 463 individuals of 58 species in tree plantations and 628 individuals of 31 species in urban settlements. Among the unclassified species of the regional pool, we found 35 species in CP, 11 in TP, 7 in US and 18 in CF. In CP and CF the most abundant species were related to rural and open habitats (as grasslands or wetlands), such as *Vanellus chilensis* (Southern Lapwing), *Guira guira* (Guira Cuckoo), *Sturnella superciliaris* (White-browed Meadowlark), *Ammodramus humeralis* (Grassland Sparrow), and *Rhynchotus rufescens* (Red-winged Tinamou). The House sparrow was dominant in US (see Supplementary Table S3).

In the Grassland, all habitat types showed similar mean functional diversity, except for US, which had the lowest functional mean mpd. Urban settlements and CP exhibited the lowest and the highest phylogenetic mean mpd, respectively. In the Forest, CF showed the lowest functional and phylogenetic mean mpd, and TP and NH the highest ones, respectively (Table 1).

**Table 1:** Functional and phylogenetic mpd mean and standard error (SE) values in each habitat type from Grassland and Forest.

	CF	US	TP	CP	NH
**Functional mpd**					
Grassland					
Mean	0.517	0.317	0.539	0.546	0.569
SE	0.019	0.042	0.019	0.019	0.027
Forest					
Mean	0.402	0.509	0.623	0.526	0.561
SE	0.031	0.027	0.024	0.024	0.031
**Phylogenetic mpd**					
Grassland					
Mean	129.6	87.4	129.8	133.5	116.9
SE	5.9	5.9	5.9	5.9	8.3
Forest					
Mean	91.8	102.1	114.5	121.1	139.2
SE	6.6	5.7	5.1	5.1	6.6

*CF* crop fields, *US* urban settlements, *TP* tree plantations, *CP* cattle pastures, *NH* natural habitats.

**Figure 2:**
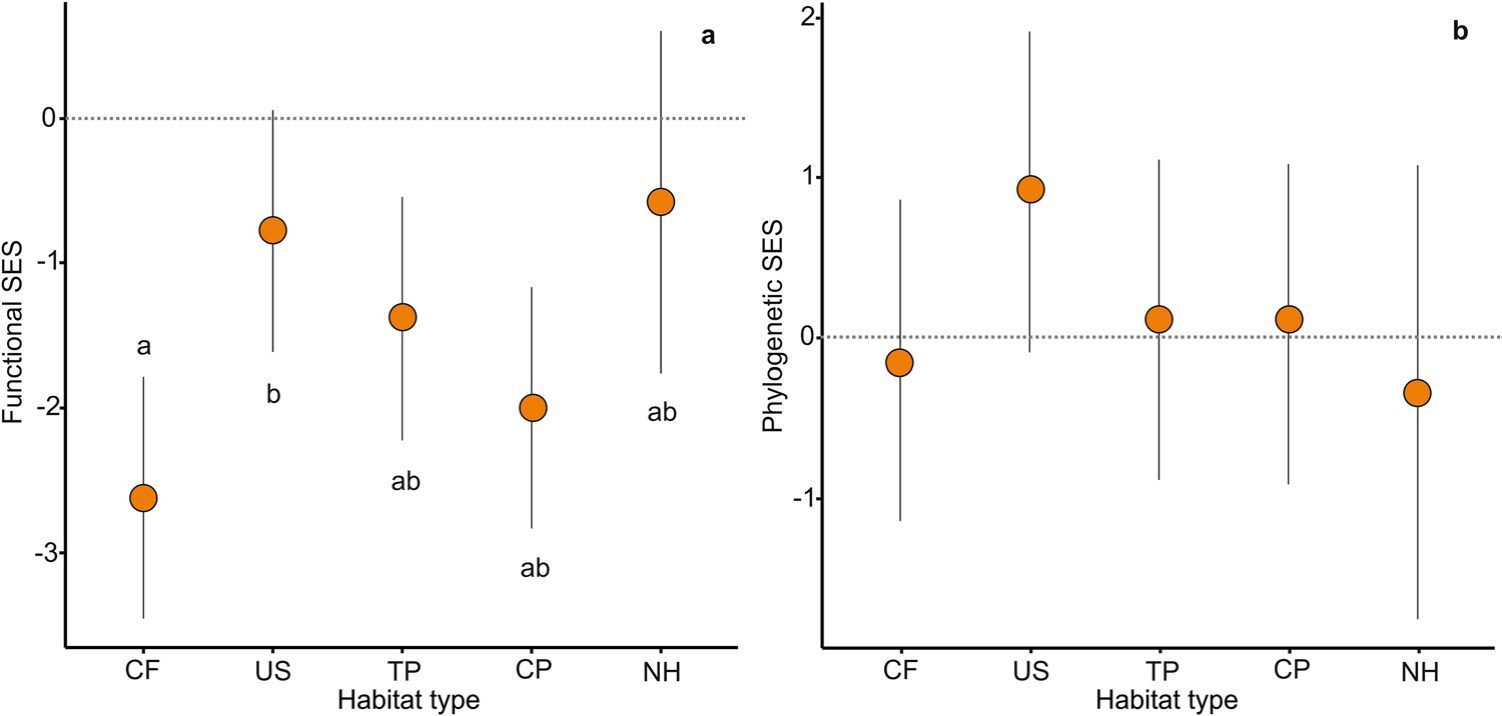
Differences in functional (**a**) and phylogenetic (**b**) SES among habitat types (*CF* crop fields, *US* urban settlements, *TP* tree plantations, *CP* cattle pastures, *NH* natural habitats) in the Grassland. Circles are means and bars are equivalent to confidence intervals of 95%. Different letters indicate significant differences (*P* < 0.05) between pairs of habitat types: results of Tukey multiple comparisons of means through emmeans function of functional and phylogenetic SES between habitat types.

In the Grassland, the functional SES values of all habitat types were significantly lower than zero, except for those of NH and US (Fig. 2a, Supplementary Table S4), indicating that species are functionally more similar than expected by chance. Although functional SES values in NH and US showed a negative trend, they did not differ significantly from zero. When performing pairwise comparisons among all habitat types, CF resulted functionally more redundant than US as its functional SES was significantly lower than the latter. Urban settlements, TP, CP, and NH were similar as Tukey tests showed no significant differences among them (Fig. 2a, Supplementary Table S5). On the other hand, the phylogenetic SES of all habitat types in the Grassland, even the positive trend of US, did not differ significantly from zero (Fig. 2b, Supplementary Table S6), that is phylogenetic structures did not differ from random expectations.

**Figure 3:**
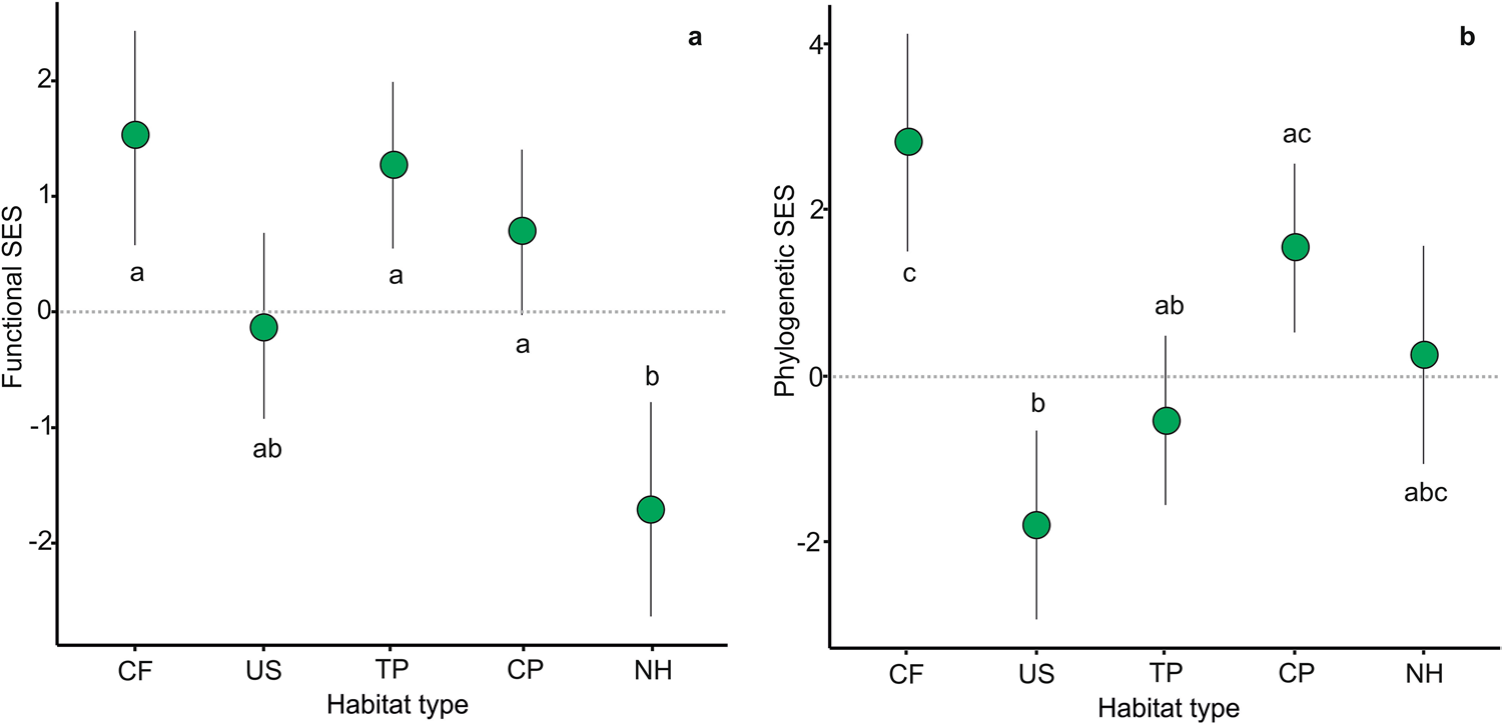
Differences in functional (**a**) and phylogenetic (**b**) SES among habitat types (*CF* crop fields, *US* urban settlements, *TP* tree plantations, *CP* cattle pastures, *NH* natural habitats) in the Forest. Circles are means and bars are equivalent to confidence intervals of 95%. Different letters indicate significant differences (*P* < 0.05) between pairs of habitat types: results of Tukey multiple comparisons of means through emmeans function of functional and phylogenetic SES between habitat types.

In the Forest, functional SES was significantly lower than zero only in NH, showing functional redundancy, and significantly positive in CF and TP, indicating functional divergence. Functional SES of CP and US did not differ significantly from zero (Fig. 3a and Supplementary Table S8). When comparing habitat types, NH showed the lowest functional mean SES, which was significantly different from those of CP, TP and CF. The functional SES of CP, TP, CF and US did not differ significantly from each other (Fig. 3a and Supplementary Table S9). On the other hand, results of phylogenetic SES, based in 95% CIs and Tukey comparisons, showed that bird communities in CF and CP had greater phylogenetic distance between co-occurring species than expected by chance, while US showed phylogenetic clustering (Fig. 3b, Supplementary Tables S10 and S11). Phylogenetic SES of NH and TP did not differ from zero, thus phylogenetic structures did not differ from random expectations.

## Discussion

Our results revealed that factors involved in bird community assembly in anthropogenic habitats depend on the biome in which they are located. In Grassland, most anthropogenic habitat types showed functional redundancy, suggesting that environmental filtering was the main driver for community assembly. Conversely, functional divergence was the dominant pattern in the Forest, pointing to competitive exclusion as the major driving factor. As we expected, we found a tendency (albeit not significant) toward greater functional redundancy in open habitats in the Grassland. In the Forest, contrary to our expectations, these habitat types showed functional divergence. Although functional divergence was not found in urban settlements and tree plantations in the Grassland, there was a tendency toward low functional redundancy. In Grassland, functional patterns do not appear to be associated with phylogenetic structures that reflect an evolutionary pattern of traits. In the Forest, functional divergence was probably caused by evolutionary trait convergence in crop fields and cattle pastures, and trait conservation in urban settlements.

In the Grassland, bird assemblages in cattle pastures, tree plantations and crop fields showed functional redundancy, while a tendency toward this pattern was observed in urban settlements and natural habitats. These results may indicate that environmental filtering was the primary driver of community assembly, and possibly even in natural habitats. Similar results were reported by Concepción et al.^48^, who proposed that the long-term land-use history in Switzerland contributed to filter out breeding birds with strong nesting-site fidelity. It is possible that the highly disturbed habitats in the Pampean region prevent the occurrence of grassland specialists^49,50^. We assume that that the current regional pool of bird species in the Pampean grasslands is the result of environmental filters caused by land-use changes since the seventeenth century^51^, when livestock was first introduced from Europe. Filloy and Bellocq^52^ found that many bird species in the Pampean region showed no response to the spatial agricultural gradient suggesting that they were tolerant to changes in land-use over time. However, in the present study functional redundancy was lower in urban settlements and, less evident, in tree plantations than in open habitats. Such result is consistent with what we expected, as these habitats include new resources such as shrubs, trees, flower plants and buildings. These provide a higher variety of food items and nesting substrates favoring the presence of species with traits different from those of species adapted to open habitats. This is in agreement with the study of Concepcion et al.^48^ in urban environments and supports our hypothesis that the incorporation of structural or environmental features into the native habitat favors the occurrence of new species with different traits. These are typically characterized by having a wide range of requirements^53,54^ and do not necessarily belong to the regional species pool.

The habitat types of the Grassland showed random phylogenetic structures, suggesting that the formation of bird assemblages was not related to evolutionary conservation or convergence of characters. However, assemblies formed by environmental filtering are expected to exhibit phylogenetic clustering or evenness^55^. It is possible that, on the one hand, environmental filtering may have selected for species with traits inherited from a common ancestor (e.g. birds of the families Rheidae and Tinamidae, which use exclusively ground and herbaceous stratum). On the other hand, phylogenetically distant species sharing similar traits may have also been selected (e.g. granivorous and ground-foraging birds of the families Emberizidae, Thraupidae, Columbidae and Psittacidae). Cavender-Bares et al.^20^ stated that environmental filtering may cause phylogenetic clustering if the ecologically important trait is highly conserved, but it may also drive phylogenetic overdispersion if other traits important to habitat specialization are labile. Therefore, despite our results indicated random phylogenetic assembly of species, phylogenetic structures may have resulted from a combination of processes.

In the Forest we found evidence of functional divergence in bird assemblages of tree plantations and crop fields and, less evident, of cattle pastures. This indicates a prevalence of competitive exclusion driving community assembly^47^. Our results could be due to the predominance of strong competitors bearing novel characteristics. Open habitats, for example, can act as novel ecosystems^56^ allowing the presence of extra-regional species with different traits. Surprisingly, bird assemblages in the native forests (i.e., natural habitats of the Forest) were characterized by functional redundancy. These results are in accordance with those of Vaccaro and Bellocq^11^, who, for these same sites, found lower and higher functional diversity in natural habitats and tree plantations, respectively, than in other habitat types. Tree plantations exhibited functional divergence probably because they harbored not only species with traits similar to those inhabiting the native forest^57^, but also generalist species that forage and nest in a variety of environments^11^. Tree plantations are generally unsuitable for native forest birds^58,59^, while extra-regional species possess traits that enable them to take advantage of the resources available not only in these habitat types but also in open habitats.

Our results in the Forest may reflect evolutionary trait convergence in crop fields and cattle pastures and evolutionary trait conservation in urban settlements and, less evident, in tree plantations. The combined results of functional divergence and phylogenetic overdispersion in open habitats seem to be consistent with the evolutionary conservation of traits associated with niche occupancy. This suggests the predominance of species interactions (competitive exclusion) over environmental filtering in the process of community assembly. García-Navas and Thuiller^26^ proposed that farmland bird assemblages are composed of very dissimilar species because limiting similarity precludes the co-existence of functionally redundant taxa. Phylogenetic overdispersion could be associated with environmental filtering if ecologically relevant traits are phylogenetically convergent^55^. Therefore, the bird assemblage found in crop fields and cattle pastures in the Forest may also reflect an early stage of environmental filtering in which functional redundancy would become apparent with time. In the case of tree plantations, the combined result of functional divergence and random phylogenetic structure with a tendency toward clustering may be associated with competitive exclusion if there had been evolutionary convergence^55^; it means that there are species with different traits because they are strong competitors. Finally, urban settlements revealed a random functional pattern but a clustering phylogenetic structure. If we assume that phylogeny represents functional differences in species assemblage composition^60^ and urbanization leads to the loss of traits^11,61^, then phylogenetic clustering results from environmental filtering selection of closely related species that share similar niches^62,63^. The study of Sol et al.^64^ reported that exotic species did not compensate for the loss of phylogenetic diversity related to urbanization. Since we did not detect functional redundancy to support environmental filtering, phylogenetic clustering may be a result of competitive exclusion^20^. In conclusion, except for urban settlements, competitive exclusion seems to play a more relevant role than environmental filtering in the process of bird assembly in anthropogenic habitats within the Forest; this process appeared to be more evident in the open habitats, which may act as sinks of novel, highly competitive species.

The prevalences of functional redundancy and functional divergence patterns and their associated main mechanism of community assembly depend on the biome and the regional species pool. In contrast to previous studies showing that land uses lead to functional redundancy^7,65,66^, here we provide evidence that the predominant pattern (and its associated mechanism of community assembly) depends on the biome and the regional species pool. For example, bird assemblages in crop fields or cattle pastures exhibited functional redundancy in one context (grassland) but functional divergence in another (forest). In the Grassland, species of the regional pool may be characterized as using open habitats and being able to coexist with each other due to the long-term history of land use in the region. In the Forest, for example, open habitats allow the presence of species with different traits and belonging to different phylogenetic lineages that did not occur before in the biome. Prevalences of functional redundancy or divergence do not ensure that the process of community assembly was driven by environmental filtering or competitive exclusion. In fact, these patterns could be attributed to new environments where assemblages are still in an early stage of development. For instance, although in most anthropogenic habitat types in the Forest we found functional divergence, this pattern could be associated with competitive exclusion or an early stage of environmental filtering in assemblage formation. Finally, it is worth noting that functional redundancy or divergence do not necessarily imply a worse or better situation for the conservation of native bird species, respectively. Regional contexts, traits and phylogenetic structures associated with these patterns must be considered for a deeper understanding of the factors driving community assembly in anthropogenic habitats.

## Supplementary Information

Supplementary Information 1.

Supplementary Information 2.

## Data Availability

The datasets used and analysed during the current study are available in the supplementary information files.
