# Supplementary material for: Factors underlying bird community assembly in anthropogenic habitats depend on the biome
Source: Sci Rep. 2022 Nov 17;12:19804. doi: 10.1038/s41598-022-24238-x (PMC9672092; doi:10.1038/s41598-022-24238-x)
Supplement: Supplementary file 1 — Supplementary Information 1. [file 41598_2022_24238_MOESM1_ESM.pdf]

Factors underlying bird community assembly in anthropogenic habitats depend on the biome

ANAHÍ S. VACCARO[a]*, JULIETA FILLOY[a]

[a]Departamento de Ecología, Genética y Evolución, IEGEBA (CONICET-UBA), Facultad de Ciencias Exactas y Naturales, Universidad de Buenos Aires, Ciudad Universitaria, Pabellón 2, Piso 4, CA Buenos Aires (C1428EGA), Argentina.

*anahivaccaro@ege.fcen.uba.ar

SUPPLEMENTARY INFORMATION

Supplementary Table S1: Bird traits used in this study

Main group/TRAIT TYPE		Abbreviation	Description and references
Feeding/DIET	Frugivore	DIET_Frug	This trait indicates position in food web and provides information on ecosystem services (e.g., population regulation of invertebrate and vertebrate species, pollination and seed dispersal).<br><br>Sekercioglu 2006, Cofre et al. 2007, Petchey et al. 2007, Flynn et al. 2009, Vandewalle et al. 2010, Luck et al. 2012, Leveau 2013, Newbold et al. 2014, Corbelli et al. 2015.
	Granivore	DIET_Gran	
	Carnivore	DIET_Carn	
	Carrion eater	DIET_Carr	
	Herbivore	DIET_Herb	
	Insectivore	DIET_Insec	
	Non-insect invertebrates	DIET_Inver	
	Omnivore	DIET_Omn	
	Nectarivore	DIET_Nect	
Feeding/FORAGING substrate	Ground	FOR_Grou	This trait indicates where foraging takes place. Species with particular foraging behavior might be impacted by environmental change.<br><br>Martin and Possingham 2005, Petchey et al. 2007, Flynn et al. 2009, Luck et al. 2012, Leveau 2013, Corbelli et al. 2015, Salgado-Negret and Paz 2016.
	Shrub	FOR_Shru	
	Tree	FOR_Tree	
	Air	FOR_Air	
	Water	FOR_Wat	
	Foraging all over	FOR_All	
	Ground	FOR_Grou	
	Shrub	FOR_Shru	
Feeding/BODY size	Less than 100 g	BODY_Small	Body size is strongly related to a range of other traits (e.g., metabolic rate, foraging behavior, longevity and home-range size).<br><br>Cofre et al. 2007, Feeley et al. 2007, Petchey et al. 2007, Flynn et al. 2009, Vandewalle et al. 2010, Luck et al. 2012, Corbelli et al. 2015, Salgado-Negret and Paz 2016.
	100-500 g	BODY_Med	
	More than 500 g	BODY_Larg	

Breeding/CLUTCH size	One egg	CLUT_Small	Species with low reproductive rates (e.g., small clutch size, infrequent breeding and low annual productivity) and low survival rates are less resilient to environmental change (i.e. reduced capacity to recover from disturbances) than those with high reproductive rates.<br><br>Cofre et al. 2007, Petchey et al. 2007, Luck et al. 2012, Corbelli et al. 2015.
	Two to three eggs	CLUT_Med	
	More than three eggs	CLUT_Larg	
Breeding/NESTING habitats	Ground	NEST_Grou	This trait indicates sensitivity to different habitat changes that affect the nesting habitats' availability. It is related to the engineering of ecosystems because, for example, it involves the construction of nests that are later used by many other organisms.<br><br>Sekercioglu 2006, Vandewalle et al. 2010, Wenny et al. 2011, Luck et al. 2012
	Water	NEST_Wat	
	Grass	NEST_Gras	
	Shrub	NEST_Shru	
	Tree	NEST_Tree	
	Natural cavity	NEST_NatCav	
	Other species' nest	NEST_Other	
	Brood parasite	NEST_Par	
	Nesting generalist	NEST_Gener	
	Buildings or other human-made structures	NEST_Build	
Breeding/MIGRATORY status	Resident	MS_R	This trait might influence large-scale cycling of nutrients and the delivery of services across broad regions and determines seasonal change in community composition. Categories described in Narosky and Yzurieta (2006).<br><br>Cofre et al. 2007, López-Lanús et al. 2008, Luck et al. 2012, Leveau 2013, Corbelli et al. 2015.
	Migratory A	MS_A	
	Migratory B	MS_B	

	Migratory C	MS_C	
Habitat/NUMBER OF HABITATS USED	One to three	NHU_Small	Habitat generalists are more resilient than specialists to environmental change because they can choose among a variety of habitats. This trait is also related to the number of different habitats in which birds fulfill ecosystem functions.<br><br>López-Lanús et al. 2008, Luck et al. 2012, Corbelli et al. 2015
	More than three	NHU_Big	
Habitat/Primary HABITAT	Forest	HAB_Forest	This trait is closely related to the principal habitat where the different species are found. It includes human-modified habitats.<br><br>Stotz et al. 1996, Cofre et al. 2007, Feeley et al. 2007, López-Lanús et al. 2008, Vandewalle et al. 2010, Corbelli et al. 2015
	Shrub	HAB_Shru	
	Urban and rural areas	HAB_Rur	
	Urban area	HAB_Urb	
	Wetland	HAB_Wet	
	Grassland	HAB_Grass	
	No primary habitat	HAB_Many	
Vulnerability/ SENSITIVITY to human disturbance	Favorable	SENS_Fav	This trait is related to the way in which the different species react to anthropogenic processes of environmental modification, replacement and pollution. Some of them benefit from human disturbance, while others show low, medium or high sensitivity.<br><br>Stotz et al. 1996, López-Lanús et al. 2008, Corbelli et al. 2015
	Low	SENS_Low	
	Medium	SENS_Med	
	High	SENS_High	
Vulnerability/ ABUNDANCE	Common	ABUND_Com	Population abundance is strongly related to the species' risk of extinction.<br><br>Stotz et al. 1996, Cofre et al. 2007, Feeley et al. 2007, López-Lanús et al. 2008
	Frequent	ABUND_Freq	
	Limited	ABUND_Lim	
Vulnerability/ DISTRIBUTION	Cosmopolitan	DIST_Cosm	Species with very restricted distributions have higher risk of extinction than those with broader ones. It is also related to the
	Neotropical	DIST_Neo	

	South America	DIST_SouAme	regional breadth of the ecosystem functions of each species.<br><br>Gillespie 2002, Sekercioglu et al. 2004, Cofre et al. 2007, Feeley et al. 2007, López-Lanús et al. 2008
	Southern South America	DIST_South	

Supplementary Table S2: Table of species and abundance per habitat type in the Grassland (NH: natural habitats, CP: cattle pastures, TP: tree plantations, US: urban settlements, CF: crop fields). Scientific names, common names and families are shown. The taxonomic order proposed by Remsen et al. (2020) was used. Species with an asterisk (*) are not considered from the regional pool.

**Scientific name**	**Common name**	**Family**	**NH**	**CP**	**TP**	**US**	**CF**
Rhea americana	Greater rhea	Rheidae	1	0	0	0	0
Rhynchotus rufescens	Red-winged tinamou	Tinamidae	18	0	6	0	1
Nothura maculosa	Spotted nothura	Tinamidae	2	5	0	0	14
Chauna torquata	Southern screamer	Anhimidae	0	2	0	0	0
Dendrocygna viduata	White-faced whistling-duck	Anatidae	1	6	0	0	0
Amazonetta brasiliensis	Brazilian teal	Anatidae	2	0	0	0	0
Anas sibilatrix	Chiloe wigeon	Anatidae	1	0	0	0	0
Anas flavirostris	Yellow-billed teal	Anatidae	0	3	0	0	0
Casmerodius albus	Great egret	Ardeidae	16	0	0	0	0
Syrigma sibilatrix	Whistling heron	Ardeidae	0	2	1	0	0
Egretta thula	Snowy egret	Ardeidae	0	6	0	0	0
Plegadis chihi	White-faced ibis	Threskiornithidae	0	55	0	0	2
Phimosus infuscatus	Bare-faced ibis	Threskiornithidae	0	6	0	0	0
Cathartes aura	Turkey vulture	Cathartidae	0	0	2	0	0
Rostrhamus sociabilis	Snail kite	Accipitrinae	0	1	0	0	0
Circus cinereus	Cinereous harrier	Accipitrinae	1	0	0	0	0
Circus buffoni	Long-winged harrier	Accipitrinae	0	0	0	0	1
Buteo magnirostris	Roadside hawk	Accipitrinae	5	0	14	0	1
Aramus guarauna	Limpkin	Aramidae	0	0	1	0	0
Vanellus chilensis	Southern lapwing	Charadriidae	19	219	16	6	95
Himantopus mexicanus	Black-necked stilt	Recurvirostridae	0	3	0	0	0
Gallinago paraguaiae	South american snipe	Scolopacidae	0	1	0	0	0
Larus maculipennis	Brown-hooded gull	Laridae	0	34	0	6	0
*Columba livia**	Rock pigeon	Columbidae	0	0	0	83	0
Patagioenas picazuro	Picazuro pigeon	Columbidae	6	4	64	6	11
Patagioenas maculosa	Spot-winged pigeon	Columbidae	1	1	0	31	0
*Leptotila verreauxi**	White-tipped dove	Columbidae	0	0	11	0	0
Zenaida auriculata	Eared dove	Columbidae	82	81	26	321	21
Guira guira	Guira cuckoo	Cuculidae	1	9	0	0	10
Tapera naevia	Striped cuckoo	Cuculidae	8	0	0	0	0
Athene cunicularia	Burrowing owl	Strigidae	0	7	0	0	4
Podager nacunda	Nacunda nighthawk	Caprimulgidae	0	2	0	0	1
Chlorostilbon lucidus	Glittering-bellied emerald	Trochilidae	0	0	1	5	0
Hylocharis chrysura	Gilded hummingbird	Trochilidae	0	0	2	0	0
*Melanerpes candidus**	White woodpecker	Picidae	0	0	1	0	0
*Colaptes melanochloros**	Green-barred woodpecker	Picidae	0	0	3	0	0

Supplementary Table S2: Continued.

Scientific name	Common name	Family	NH	CP	TP	US	CF
Colaptes campestris	Field flicker	Picidae	2	7	0	0	2
Caracara plancus	Southern caracara	Falconidae	0	4	1	0	2
Milvago chimango	Chimango caracara	Falconidae	12	15	19	5	13
Falco sparverius	American krestel	Falconidae	1	3	0	0	0
Myiopsitta monachus	Monk parakeet	Psittacidae	2	46	12	1	40
*Thamnophilus caerulescens**	Variable antshrike	Thamnophilidae	0	0	1	0	0
*Lepidocolaptes angustirostris**	Narrow-billed woodcreeper	Furnariidae	3	0	4	0	0
Furnarius rufus	Rufous hornero	Furnariidae	3	29	16	51	23
Phacellodomus striaticollis	Freckle-breasted thornbird	Furnariidae	13	1	0	0	1
Anumbius annumbi	Firewood-gatherer	Furnariidae	0	1	0	0	1
Synallaxis albescens	Pale-breasted spinetail	Furnariidae	0	1	0	0	0
*Synallaxis frontalis**	Sooty-fronted spinetail	Furnariidae	1	0	0	0	0
*Camptostoma obsoletum**	Southern beardless tyrannulet	Tyrannidae	0	0	1	0	0
*Serpophaga subcristata**	White-crested tyrannnulet	Tyrannidae	0	0	5	1	1
Polystictus pectoralis	Bearded tachuri	Tyrannidae	1	0	0	0	0
Pseudocolopteryx flaviventris	Warbling doradito	Tyrannidae	2	0	0	0	0
*Myiophobus fasciatus**	Bran-colored flycatcher	Tyrannidae	0	0	1	0	5
Hymenops perspicillatus	Spectacled tyrant	Tyrannidae	2	0	0	0	0
Xolmis cinereus	Gray monjita	Tyrannidae	5	2	0	0	0
Xolmis irupero	White monjita	Tyrannidae	0	0	0	0	1
Machetornis rixosa	Cattle tyrant	Tyrannidae	1	1	0	3	1
Tyrannus savana	Fork-tailed flycatcher	Tyrannidae	18	29	0	0	11
Pitangus sulphuratus	Great kiskadee	Tyrannidae	6	25	16	34	12
*Myiodynastes maculatus**	Streaked flycatcher	Tyrannidae	0	0	3	0	0
*Tyrannus melancholicus**	Tropical kingbird	Tyrannidae	0	1	0	0	0
Pygochelidon cyanoleuca	Blue-and-white swallow	Hirundinidae	0	0	0	5	0
Progne tapera	Brown-chested martin	Hirundinidae	0	0	1	0	1
Progne chalybea	Gray-breasted Martin	Hirundinidae	0	0	4	2	0
Tachycineta leucorrhoa	White-rumped swallow	Hirundinidae	1	17	0	1	6
Hirundo rustica	Barn swallow	Hirundinidae	0	10	0	0	0
Troglodytes aedon	House wren	Troglodytidae	2	5	43	11	0
Cistothorus platensis	Sedge wren	Troglodytidae	4	0	0	0	0
*Polioptila dumicola**	Masked gnatcatcher	Polioptilidae	0	0	1	0	0
Turdus rufiventris	Rufous-bellied thrush	Turdidae	0	0	11	4	12
*Turdus amaurochalinus**	Creamy-bellied thrush	Turdidae	0	3	2	0	0
Mimus saturninus	Chalk-browed mockingbird	Mimidae	1	4	0	2	1
Anthus lutescens	Yellowish pipit	Motacillidae	0	14	0	0	0
Anthus correndera	Correndera pipit	Motacillidae	0	4	0	0	0
Anthus hellmayri	Hellmayr's pipit	Motacillidae	2	16	0	0	0
Paroaria coronata	Red-crested cardinal	Thraupidae	0	1	0	0	0
Donacospiza albifrons	Long-tailed reed finch	Thraupidae	3	0	0	0	0

Supplementary Table S2: Continued.

Scientific name	Common name	Family	NH	CP	TP	US	CF
Sicalis flaveola	Saffron yellow-finch	Thraupidae	0	0	0	0	1
Sicalis luteola	Grassland yellow-finch	Thraupidae	37	32	2	0	29
Embernagra platensis	Great pampa-finch	Thraupidae	17	0	0	0	0
*Saltator aurantiirostris**	Golden-billed saltator	Incertae sedis	7	0	0	0	0
Aimophila strigiceps	Stripe-capped sparrow	Emberizidae	1	0	0	0	0
Ammodramus humeralis	Grassland sparrow	Emberizidae	7	10	0	0	14
Zonotrichia capensis	Rufous-collared sparrow	Emberizidae	8	34	59	9	47
Parula pitiayumi	Tropical parula	Parulidae	1	0	4	0	0
Pseudoleistes virescens	Brown-and-yellow marshbird	Icteridae	2	0	0	0	0
Agelaioides badius	Grayish baywing	Icteridae	0	1	0	0	0
Molothrus rufoaxillaris	Screaming cowbird	Icteridae	0	0	0	2	0
Molothrus bonariensis	Shiny cowbird	Icteridae	2	6	0	19	0
Sturnella superciliaris	White-browed meadowlark	Icteridae	0	15	0	0	7
Carduelis magellanica	Hooded siskin	Fringillidae	2	1	6	3	0
*Passer domesticus**	House sparrow	Passeridae	0	2	0	290	0

Supplementary Table S3: Table of species and abundance per habitat type in the Forest (NH: natural habitats, CP: cattle pastures, TP: tree plantations, US: urban settlements, CF: crop fields). Scientific names, common names and families are shown. The taxonomic order proposed by Remsen et al. (2020) was used. Species with an asterisk (*) are not considered from the regional pool.

**Scientific names**	**Common names**	**Family**	**NH**	**TP**	**CP**	**CF**	**US**
*Rhea americana**	Greater Rhea	Rheidae	0	1	2	2	0
Crypturellus obsoletus	Brown Tinamou	Tinamidae	0	1	0	0	0
Crypturellus undulatus	Undulated Tinamou	Tinamidae	7	0	0	0	0
Crypturellus parvirostris	Small-billed Tinamou	Tinamidae	0	2	0	0	0
Crypturellus tataupa	Tataupa Tinamou	Tinamidae	4	1	0	0	0
*Rhynchotus rufescens**	Red-winged Tinamou	Tinamidae	0	0	25	12	0
*Nothura maculosa**	Spotted Nothura	Tinamidae	0	0	22	8	0
*Dendrocygna viduata**	White-faced Whistling-Duck	Anatidae	0	0	1	0	0
*Amazonetta brasiliensis**	Brazilian Teal	Anatidae	0	0	2	0	0
*Columba livia**	Rock Pigeon	Columbidae	0	0	0	0	17
Patagioenas picazuro	Picazuro Pigeon	Columbidae	14	9	5	3	0
Patagioenas cayennensis	Pale-vented Pigeon	Columbidae	1	0	0	0	0
Leptotila verreauxi	White-tipped Dove	Columbidae	20	7	5	0	0
Leptotila rufaxilla	Gray-fronted Dove	Columbidae	5	1	0	0	0
Zenaida auriculata	Eared Dove	Columbidae	0	5	24	11	0
Columbina talpacoti	Ruddy Ground Dove	Columbidae	1	8	0	3	0
Columbina picui	Picui Ground Dove	Columbidae	0	2	0	0	4
*Guira guira**	Guira Cuckoo	Cuculidae	0	10	8	31	2
Crotophaga ani	Smooth-billed Ani	Cuculidae	0	0	2	0	0
Piaya cayana	Squirrel Cuckoo	Cuculidae	6	1	0	0	0
*Podager nacunda**	Nacunda Nighthawk	Caprimulgidae	0	0	2	0	0
Caprimulgus parvulus	Little Nightjar	Caprimulgidae	0	0	0	0	1
Chaetura meridionalis	Sick's Swift	Apodidae	0	0	0	0	9
Chlorostilbon lucidus	Glittering-bellied Emerald	Trochilidae	0	0	0	0	2
Hylocharis chrysura	Gilded Hummingbird	Trochilidae	0	8	0	0	0
*Porzana albicollis**	Ash-throated Crake	Rallidae	0	0	5	0	0
*Gallinula chloropus**	Common Gallinule	Rallidae	0	0	1	0	0
*Vanellus chilensis**	Southern Lapwing	Charadriidae	0	10	56	18	12
*Himantopus mexicanus**	Black-necked Stilt	Recurvirostridae	0	0	1	0	0
*Bartramia longicauda**	Upland Sandpiper	Scolopacidae	0	0	2	5	0
*Jacana jacana**	Wattled Jacana	Jacanidae	0	0	2	0	0
*Bubulcus ibis**	Cattle Egret	Ardeidae	0	14	15	11	0
*Syrigma sibilatrix**	Whistling Heron	Ardeidae	0	1	1	1	0
Coragyps atratus	Black Vulture	Cathartidae	0	0	16	0	1
*Circus buffoni**	Long-winged Harrier	Accipitridae	0	0	2	6	0
Buteo magnirostris	Roadside Hawk	Accipitridae	1	6	0	0	0

Supplementary Table S3: Continued.

Scientific names	Common names	Family	NH	TP	CP	CF	US
Geranoaetus melanoleucus	Black-chested Buzzard-Eagle	Accipitridae	0	1	0	0	0
Glaucidium brasilianum	Ferruginous Pygmy-Owl	Strigidae	1	2	0	0	0
*Athene cunicularia**	Burrowing Owl	Strigidae	0	0	10	5	0
Trogon surrucura	Surucua Trogon	Trogonidae	6	5	0	0	0
Trogon rufus	Black-throated Trogon	Trogonidae	1	0	0	0	17
Baryphthengus ruficapillus	Rufous-capped Motmot	Momotidae	3	0	0	0	0
*Megaceryle torquata**	Ringed Kingfisher	Alcedinidae	0	0	1	0	0
Nystalus chacuru	White-eared Puffbird	Bucconidae	0	4	1	0	0
Ramphastos dicolorus	Red-breasted Toucan	Ramphastidae	1	0	0	0	0
Selenidera maculirostris	Spot-billed Toucanet	Ramphastidae	1	0	0	0	0
Pteroglossus castanotis	Chestnut-eared Aracari	Ramphastidae	5	0	0	0	0
Melanerpes candidus	White Woodpecker	Picidae	0	0	0	0	1
*Colaptes campestris**	Campo Flicker	Picidae	0	6	32	1	0
Caracara plancus	Southern Caracara	Falconidae	0	0	5	1	0
Milvago chimachima	Yellow-headed Caracara	Falconidae	0	0	1	0	0
Milvago chimango	Chimango Caracara	Falconidae	0	0	1	1	0
Falco sparverius	American Kestrel	Falconidae	0	0	2	1	0
*Myiopsitta monachus**	Monk Parakeet	Psittacidae	0	0	2	0	0
Brotogeris chiriri	Yellow-chevroned Parakeet	Psittacidae	13	9	0	0	0
Amazona aestiva	Turquoise-fronted Parrot	Psittacidae	0	0	1	0	0
Pyrrhura frontalis	Maroon-bellied Parakeet	Psittacidae	9	0	0	0	0
Aratinga leucophthalma	White-eyed Parakeet	Psittacidae	5	4	1	0	0
Hypoedaleus guttatus	Spot-backed Antshrike	Thamnophilidae	4	0	0	0	0
Thamnophilus caerulescens	Variable Antshrike	Thamnophilidae	4	0	0	0	0
Dysithamnus mentalis	Plain Antvireo	Thamnophilidae	14	0	0	0	0
Herpsilochmus rufimarginatus	Rufous-winged Antwren	Thamnophilidae	10	0	0	0	0
Drymophila malura	Dusky-tailed Antbird	Thamnophilidae	1	0	0	0	0
Pyriglena leucoptera	White-shouldered Fire-eye	Thamnophilidae	2	0	0	0	0
Conopophaga lineata	Rufous Gnateater	Conopophagidae	1	0	0	0	0
Sittasomus griseicapillus	Olivaceous Woodcreeper	Furnariidae	1	1	0	0	0
Dendrocolaptes platyrostris	Planalto Woodcreeper	Furnariidae	2	0	0	0	0
*Furnarius rufus**	Rufous Hornero	Furnariidae	0	12	11	7	35
Philydor lichtensteini	Ochre-breasted Foliage-gleaner	Furnariidae	2	0	0	0	0
*Anumbius annumbi**	Firewood-gatherer	Furnariidae	0	0	2	1	0
Certhiaxis cinnamomeus	Yellow-chinned Spinetail	Furnariidae	0	0	4	0	0
Synallaxis cinerascens	Gray-bellied Spinetail	Furnariidae	2	0	0	0	0
Synallaxis ruficapilla	Rufous-capped Spinetail	Furnariidae	3	0	0	0	0
Myiopagis caniceps	Gray Elaenia	Tyrannidae	0	1	0	0	0
Elaenia flavogaster	Yellow-bellied Elaenia	Tyrannidae	0	0	3	0	0

Supplementary Table S3: Continued.

Scientific names	Common names	Family	NH	TP	CP	CF	US
Elaenia spectabilis	Large Elaenia	Tyrannidae	0	0	2	0	0
Serpophaga subcristata	White-crested Tyrannulet	Tyrannidae	0	1	0	0	1
Capsiempis flaveola	Yellow Tyrannulet	Tyrannidae	1	0	0	0	0
Corythopis delalandi	Southern Antpipit	Tyrannidae	1	0	0	0	0
Leptopogon amaurocephalus	Sepia-capped Flycatcher	Tyrannidae	1	0	0	0	0
*Culicivora caudacuta**	Sharp-tailed Tyrant	Tyrannidae	0	0	1	0	0
Myiornis auricularis	Eared Pygmy-Tyrant	Tyrannidae	2	0	0	0	0
Myiophobus fasciatus	Bran-colored Flycatcher	Tyrannidae	0	0	2	0	0
Lathrotriccus euleri	Euler's Flycatcher	Tyrannidae	2	0	0	0	0
Cnemotriccus fuscatus	Fuscous Flycatcher	Tyrannidae	3	0	0	0	0
*Machetornis rixosa**	Cattle Tyrant	Tyrannidae	0	2	0	0	0
Pitangus sulphuratus	Great Kiskadee	Tyrannidae	3	50	16	1	31
Conopias trivirgatus	Three-striped Flycatcher	Tyrannidae	2	0	0	0	0
Myiodynastes maculatus	Streaked Flycatcher	Tyrannidae	1	34	0	0	0
Basileuterus leucoblepharus	White-browed Warbler	Parulidae	9	0	0	0	0
Megarynchus pitangua	Boat-billed Flycatcher	Tyrannidae	11	26	0	0	0
Empidonomus varius	Variegated Flycatcher	Tyrannidae	0	4	0	0	0
Tyrannus melancholicus	Tropical Kingbird	Tyrannidae	0	11	0	0	0
*Tyrannus savana**	Fork-tailed Flycatcher	Tyrannidae	0	5	9	3	13
Sirystes sibilator	Sibilant Sirystes	Tyrannidae	1	0	0	0	0
Myiarchus ferox	Short-crested Flycatcher	Tyrannidae	0	0	1	0	0
Procnias nudicollis	Bare-throated Bellbird	Cotingidae	4	0	0	0	0
Chiroxiphia caudata	Swallow-tailed Manakin	Pipridae	1	0	0	0	0
Tityra inquisitor	Black-crowned Tityra	Tityridae	2	0	0	0	52
Schiffornis virescens	Greenish Schiffornis	Tityridae	3	0	0	0	0
Pachyramphus polychopterus	White-winged Becard	Tityridae	1	0	1	0	0
Cyclarhis gujanensis	Rufous-browed Peppershrike	Vireonidae	3	2	5	0	0
Hylophilus poicilotis	Rufous-crowned Greenlet	Vireonidae	2	0	0	0	0
Vireo olivaceus	Red-eyed Vireo	Vireonidae	1	3	0	10	4
Cyanocorax cyanomelas	Purplish Jay	Corvidae	0	4	0	0	0
Cyanocorax chrysops	Plush-crested Jay	Corvidae	6	4	0	0	0
Pygochelidon cyanoleuca	Blue-and-white Swallow	Hirundinidae	0	0	5	0	3
Progne tapera	Brown-chested Martin	Hirundinidae	0	0	3	3	1
Progne chalybea	Gray-breasted Martin	Hirundinidae	0	0	8	0	9
Tachycineta leucorrhoa	White-rumped Swallow	Hirundinidae	0	0	0	1	0
Troglodytes aedon	House Wren	Troglodytidae	8	49	6	1	0
Turdus leucomelas	Pale-breasted Thrush	Turdidae	13	49	0	0	0
Turdus rufiventris	Rufous-bellied Thrush	Turdidae	2	8	0	0	4
Turdus amaurochalinus	Creamy-bellied Thrush	Turdidae	0	0	4	0	0
*Mimus saturninus**	Chalk-browed Mockingbird	Mimidae	0	0	1	0	2
*Passer domesticus**	House Sparrow	Passeridae	0	0	0	0	320

Supplementary Table S3: Continued.

Scientific names	Common names	Family	NH	TP	CP	CF	US
*Anthus lutescens**	Yellowish Pipit	Motacillidae	0	0	16	53	0
Carduelis magellanica	Hooded Siskin	Fringillidae	0	4	0	0	3
Euphonia chlorotica	Purple-throated Euphonia	Fringillidae	1	0	0	0	3
Chlorophonia cyanea	Blue-naped Chlorophonia	Fringillidae	1	0	0	0	0
*Ammodramus humeralis**	Grassland Sparrow	Passerellidae	0	0	31	27	0
Zonotrichia capensis	Rufous-collared Sparrow	Passerellidae	0	3	8	0	34
Cacicus chrysopterus	Golden-winged Cacique	Icteridae	0	1	0	0	0
Cacicus haemorrhous	Red-rumped Cacique	Icteridae	0	2	0	0	0
Icterus cayanensis	Variable Oriole	Icteridae	4	0	0	0	5
Gnorimopsar chopi	Chopi Blackbird	Icteridae	1	12	0	0	0
*Pseudoleistes guirahuro**	Yellow-rumped Marshbird	Icteridae	0	0	8	0	0
*Pseudoleistes virescens**	Brown-and-yellow Marshbird	Icteridae	0	0	14	0	0
Agelaioides badius	Grayish Baywing	Icteridae	0	0	0	0	1
Molothrus rufoaxillaris	Screaming Cowbird	Icteridae	0	2	1	0	17
Molothrus bonariensis	Shiny Cowbird	Icteridae	0	1	5	3	1
*Sturnella superciliaris**	White-browed Meadowlark	Icteridae	0	0	52	13	0
Geothlypis aequinoctialis	Masked Yellowthroat	Parulidae	0	0	6	0	0
Parula pitiayumi	Tropical Parula	Parulidae	3	1	0	0	0
Basileuterus culicivorus	Golden-crowned Warbler	Parulidae	29	13	0	0	0
Hemithraupis guira	Guira Tanager	Thraupidae	0	2	0	0	0
Sicalis flaveola	Saffron Finch	Thraupidae	0	1	12	2	22
*Sicalis luteola**	Grassland Yellow-Finch	Thraupidae	0	1	30	1	0
*Volatinia jacarina**	Blue-black Grassquit	Thraupidae	0	2	1	0	0
Tachyphonus coronatus	Ruby-crowned Tanager	Thraupidae	1	0	0	0	0
Coryphospingus cucullatus	Red-crested Finch	Thraupidae	2	2	1	0	0
Sporophila caerulescens	Double-collared Seedeater	Thraupidae	0	0	0	0	1
Saltator coerulescens	Grayish Saltator	Thraupidae	0	0	1	0	0
Saltator aurantiirostris	Golden-billed Saltator	Thraupidae	0	2	0	0	0
*Embernagra platensis**	Great Pampa-Finch	Thraupidae	0	0	9	0	0
*Emberizoides herbicola**	Wedge-tailed Grass-Finch	Thraupidae	0	0	5	0	0
*Emberizoides ypiranganus**	Lesser Grass-Finch	Thraupidae	0	0	3	0	0
Poospiza nigrorufa	Black-and-rufous Warbling Finch	Thraupidae	0	0	1	0	0
Coereba flaveola	Bananaquit	Thraupidae	1	0	0	0	0
Thraupis sayaca	Sayaca Tanager	Thraupidae	0	30	5	0	0

Supplementary Table S4: Descriptive summary for functional SES values in the habitat types of the Grassland. It is shown the mean between functional SES values in each habitat type, standard deviations (SE) and the lower and upper confidence limits (CL) obtained with emmeans function. We used 22 Degrees-of-freedom. Confidence level used: 0.95.

Habitat types	Mean	SE	Lower CL	Upper CL
Crop fields	-2.619	0.401	-3.45	-1.7869
Urban settlements	-0.784	0.401	-1.62	0.0478
Tree plantations	-1.384	0.401	-2.22	-0.5528
Cattle pastures	-2	0.401	-2.83	-1.168
Natural habitats	-0.584	0.567	-1.76	0.592

Supplementary Table S5: Multiple comparisons of means through emmeans function for functional SES values between habitat types in the Grassland. Habitat types are urban settlements (US), cattle pastures (CP), crop fields (CF), tree plantations (TP), and natural habitats (NH). $P < 0.05$; asterisk (*) indicates significant differences.

Habitat types comparison	t value	P
CF versus US	-3.235	0.0281*
CF versus TP	-2.176	0.2255
CF versus CP	-1.091	0.8092
CF versus NH	-2.929	0.0542
US versus TP	1.059	0.8251
US versus CP	2.144	0.2378
US versus NH	-0.288	0.9984
TP versus CP	1.085	0.8123
TP versus NH	-1.152	0.7775
CP versus NH	-2.038	0.2817

Supplementary Table S6: Descriptive summary for phylogenetic SES values in the habitat types of the Grassland. It is shown the mean between functional SES values in each habitat type, standard deviations (SE) and the lower and upper confidence limits (CL) obtained with emmeans function. We used 22 Degrees-of-freedom. Confidence level used: 0.95.

Habitat types	Mean	SE	Lower CL	Upper CL
Crop fields	-0.1364	0.482	-1.1362	0.863
Urban settlements	0.9112	0.482	-0.0886	1.911
Tree plantations	0.1155	0.482	-0.8843	1.115
Cattle pastures	0.0891	0.482	-0.9107	1.089
Natural habitats	-0.335	0.482	-1.7489	1.079

Supplementary Table S7: Multiple comparisons of means through emmeans function for phylogenetic SES values between habitat types in the Grassland. Habitat types are urban settlements (US), cattle pastures (CP), crop fields (CF), tree plantations (TP), and natural habitats (NH). P < 0.05; asterisk (*) indicates significant differences.

Habitat types comparison	t value	P
CF versus US	-1.537	0.5509
CF versus TP	-0.37	0.9957
CF versus CP	-0.331	0.9972
CF versus NH	0.238	0.9992
US versus TP	1.167	0.7694
US versus CP	1.206	0.7481
US versus NH	1.492	0.5778
TP versus CP	0.039	1
TP versus NH	0.54	0.9821
CP versus NH	0.508	0.9857

Supplementary Table S8: Descriptive summary for functional SES values in the habitat types of the Forest. It is shown the mean between functional SES values in each habitat type, standard deviations (SE) and the lower and upper confidence limits (CL) obtained with emmeans function. We used 15 Degrees-of-freedom. Confidence level used: 0.95.

Habitat types	Mean	SE	Lower CL	Upper CL
Crop fields	1.501	0.435	0.5739	2.428
Urban settlements	-0.124	0.377	-0.9272	0.679
Tree plantations	1.268	0.337	0.5502	1.987
Cattle pastures	0.687	0.337	-0.0308	1.406
Natural habitats	-1.707	0.435	-2.6347	-0.78

Supplementary Table S9: Multiple comparisons of means through emmeans function for functional SES values between habitat types in the Forest. Habitat types are urban settlements (US), cattle pastures (CP), crop fields (CF), tree plantations (TP), and natural habitats (NH). P < 0.05; asterisk (*) indicates significant differences

Habitat types comparison	t value	P
CF versus US	2.824	0.0811
CF versus TP	0.423	0.9926
CF versus CP	1.479	0.5902
CF versus NH	5.216	0.0008*
US versus TP	-2.755	0.0917
US versus CP	-1.606	0.5161
US versus NH	2.751	0.0924
TP versus CP	1.219	0.7409
TP versus NH	5.408	0.0006*
CP versus NH	4.352	0.0044*

Supplementary Table S10: Descriptive summary for phylogenetic SES values in the habitat types of the Forest. It is shown the mean between functional SES values in each habitat type, standard deviations (SE) and the lower and upper confidence limits (CL) obtained with emmeans function. We used 15 Degrees-of-freedom. Confidence level used: 0.95.

Habitat types	Mean	SE	Lower CL	Upper CL
Crop fields	2.801	0.614	1.492	4.11
Urban settlements	-1.791	0.532	-2.925	-0.658
Tree plantations	-0.538	0.476	-1.552	0.475
Cattle pastures	1.533	0.476	0.519	2.547
Natural habitats	0.256	0.614	-1.053	1.565

Supplementary Table S11: Multiple comparisons of means through emmeans function for phylogenetic SES values between habitat types in the Forest. Habitat types are urban settlements (US), cattle pastures (CP), crop fields (CF), tree plantations (TP), and natural habitats (NH). $P < 0.05$; asterisk (*) indicates significant differences.

Habitat types comparison	t value	P
CF versus US	5.653	0.0004*
CF versus TP	4.299	0.0049*
CF versus CP	1.633	0.5005
CF versus NH	2.93	0.0669
US versus TP	-1.756	0.4324
US versus CP	-4.658	0.0024*
US versus NH	-2.52	0.138
TP versus CP	-3.079	0.0509
TP versus NH	-1.023	0.841
CP versus NH	1.643	0.4945

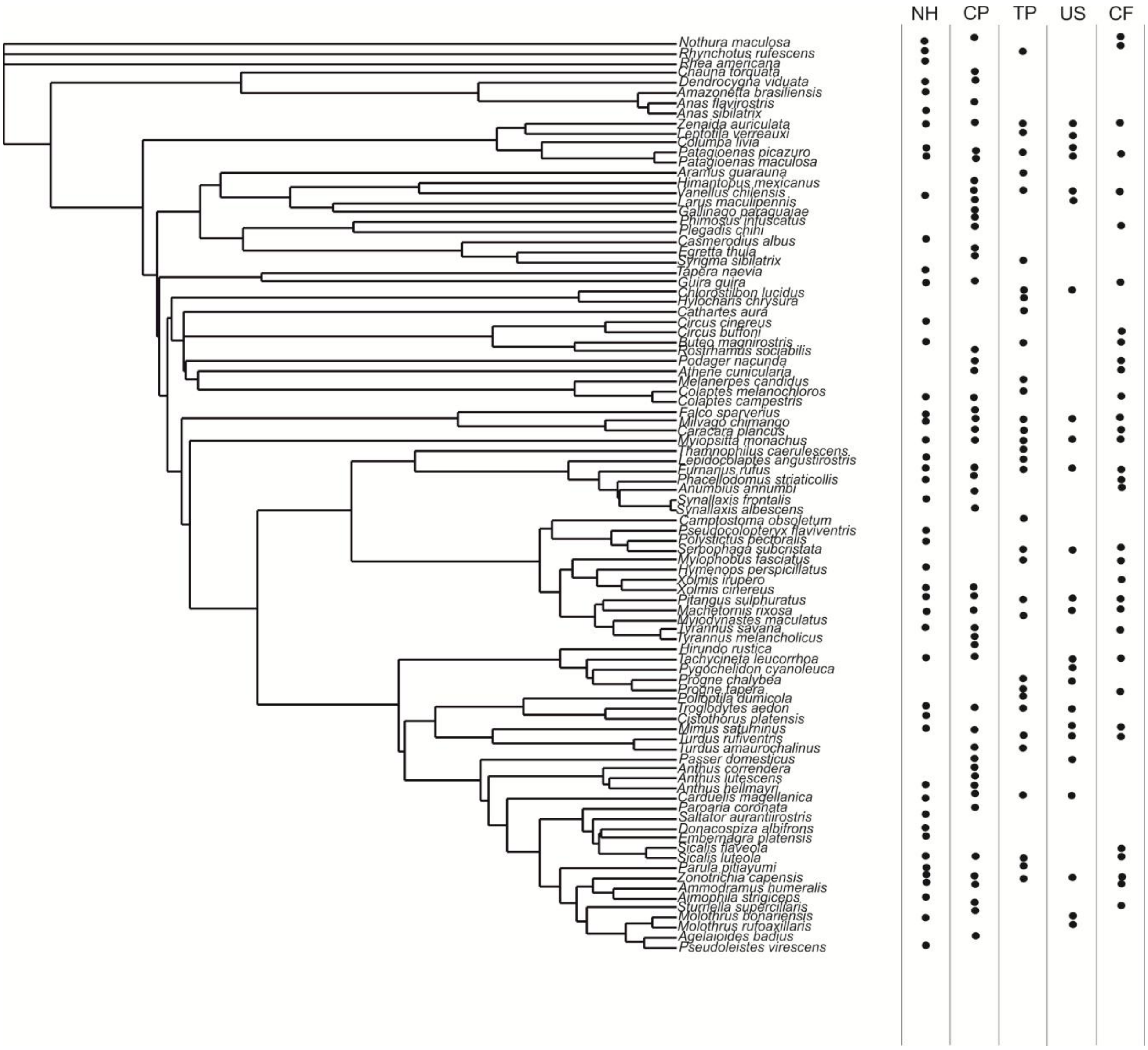

Supplementary Figure S1: Consensus phylogenetic tree of the total registered species in the Grassland. This tree was constructed with 100 extracted trees from the global phylogeny of birds (Bird Tree: birdtree.org). The presence of each species in the habitat types is shown (NH: natural habitats, CP: cattle pastures, TP: tree plantations, US: urban settlements, CF: crop fields).

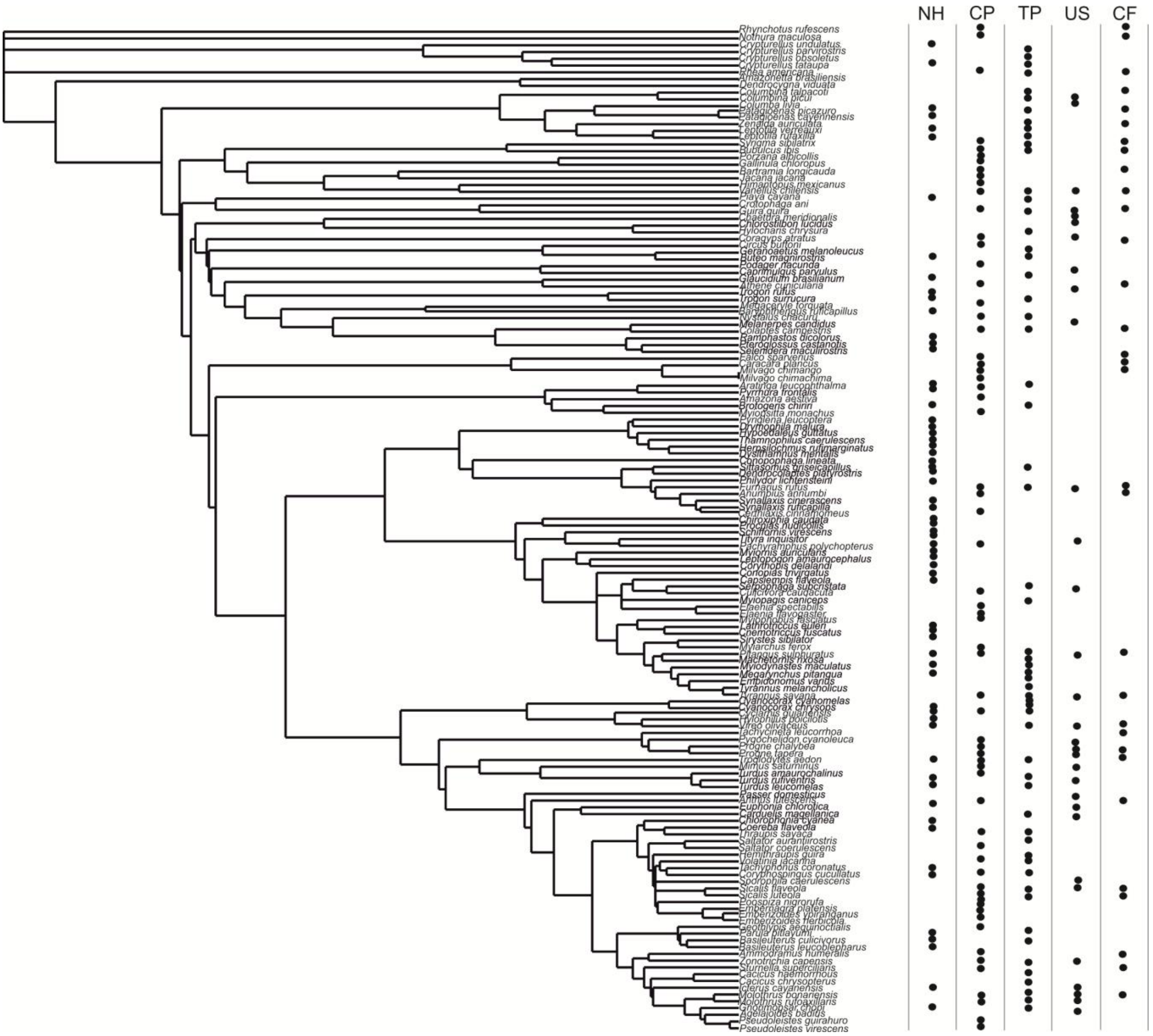

Supplementary Figure S2: Consensus phylogenetic tree of the total registered species in the Forest. This tree was constructed with 100 extracted trees from the global phylogeny of birds (Bird Tree: birdtree.org). The presence of each species in the habitat types is shown (NH: natural habitats, CP: cattle pastures, TP: tree plantations, US: urban settlements, CF: crop fields).

LITERATURE CITED

Cofre, H. L., K. Böhning-Gaese, and P. A. Marquet. Rarity in Chilean forest birds: Which ecological and life-history traits matter? Biodiversity research. *Diversity and Distributions* 13:203–212. doi: 10.1111/j.1472-4642.2006.00312.x (2007).

Corbelli, J. M., G. A. Zurita, J. Filloy, J. P. Galvis, N. I. Vespa, and I. Bellocq. Integrating taxonomic, functional and phylogenetic beta diversities: Interactive effects with the biome and land use across taxa. *PLoS ONE* 10:1–17. doi: 10.1371/journal.pone.0126854 (2015).

Feeley, K. J., T. W. Gillespie, D. J. Lebbin, and H. S. Walter. Species characteristics associated with extinction vulnerability and nestedness rankings of birds in tropical forest fragments. *Animal Conservation* 10:493–501. doi: 10.1111/j.1469-1795.2007.00140.x (2007).

Flynn, D. F. B., M. Gogol-Prokurat, T. Nogeire, N. Molinari, B. T. Richers, B. B. Lin, N. Simpson, M. M. Mayfield, and F. DeClerck. Loss of functional diversity under land use intensification across multiple taxa. *Ecology Letters* 12:22–33. doi: 10.1111/j.1461-0248.2008.01255.x (2009).

Gillespie, T. W. Latitudinal extent and natural history characteristics of birds in Nicaragua. *Global Ecology and Biogeography* 11:411–417. doi: 10.1046/j.1466-822x.2002.00295.x (2002).

Leveau, L. M. Bird traits in urban–rural gradients: how many functional groups are there? *Journal of Ornithology* 154:655–662. doi: 10.1007/s10336-012-0928-x (2013).

López-Lanús, B., P. Grilli, A. S. Di Giacomo, E. E. Coconier, and R. Banchs. *Categorización de las aves de la Argentina según su estado de conservación*. Aves Argentinas, Buenos Aires, Argentina (2008).

Luck, G. W., S. Lavorel, S. Mcintyre, and K. Lumb. Improving the application of vertebrate trait-based frameworks to the study of ecosystem services. *Journal of Animal Ecology* 81:1065–1076. doi: 10.1111/j.1365-2656.2012.01974.x (2012).

Martin, T. G., and H. P. Possingham. Predicting the impact of livestock grazing on birds using foraging height data. *Journal of Applied Ecology* 42:400–408. doi: 10.1111/j.1365-2664.2005.01012.x (2005).

Petchey, O. L., K. L. Evans, I. S. Fishburn, and K. J. Gaston. Low functional diversity and no redundancy in British avian assemblages. *Journal of Animal Ecology* 76:977–985. doi: 10.1111/j.1365-2656.2007.01271.x (2007).

Remsen Jr, J.V., J. I. Areta, E. Bonaccorso, S. Claramunt, A. Jaramillo, J. F. Pacheco, C. Ribas, M. B. Robbins, F. G. Stiles, D. F. Stotz and K. J. Zimmer. A classification of the bird species of South America. American Ornithological Society, Chicago (URL: http://www.museum.lsu.edu/~Remsen/SACCBaseline.htm) (2020).

Salgado-Negret, B. and H. Paz. Escalando de los rasgos funcionales a procesos poblacionales, comunitarios y ecosistémicos. *La ecología funcional como aproximación al estudio, manejo y conservación de la biodiversidad: protocolos y aplicaciones (B. Salgado-Negret, Editor). Instituto de Investigación de Recursos Biológicos Alexander von Humboldt*, Bogotá, Colombia, 12-35 (2015).

Sekercioglu, C. H. Increasing awareness of avian ecological function. *Trends in Ecology and Evolution* 21:464–471. doi: 10.1016/j.tree.2006.05.007 (2006).

Stotz, D. F., J. W. Fitzpatrick, T. A. Parker III, and D. K. Moskovits. 1996. Neotropical birds : ecology and conservation. Chicago, USA: *The University of Chicago Press* (1996).

Vandewalle, M., F. de Bello, M. P. Berg, T. Bolger, S. Dolédec, F. Dubs, C. K. Feld, R. Harrington, P. A. Harrison, S. Lavorel, P. M. da Silva, M. Moretti, J. Niemelä, P. Santos, T. Sattler, J. P. Sousa,

M. T. Sykes, A. J. Vanbergen, and B. A. Woodcock. Functional traits as indicators of biodiversity response to land use changes across ecosystems and organisms. *Biodiversity and Conservation* 19:2921–2947. doi: 10.1007/s10531-010-9798-9 (2010).
